# Spatiotemporal Profiles of Proprioception Processed by the Masseter Muscle Spindles in Rat Cerebral Cortex: An Optical Imaging Study

**DOI:** 10.3389/fncir.2017.00004

**Published:** 2017-01-30

**Authors:** Satoshi Fujita, Mari Kaneko, Hiroko Nakamura, Masayuki Kobayashi

**Affiliations:** ^1^Department of Pharmacology, School of Dentistry, Nihon UniversityTokyo, Japan; ^2^Division of Oral and Craniomaxillofacial Research, Dental Research Center, School of Dentistry, Nihon UniversityTokyo, Japan; ^3^Department of Orthodontics, School of Dentistry, Nihon UniversityTokyo, Japan; ^4^Department of Pediatric Dentistry, School of Dentistry, Nihon UniversityTokyo, Japan; ^5^Molecular Dynamics Imaging Unit, RIKEN Center for Life Science TechnologiesKobe, Japan

**Keywords:** insular cortex, somatosensory cortex, orofacial pain, non-odontogenic toothache, referred pain

## Abstract

Muscle spindles in the jaw-closing muscles, which are innervated by trigeminal mesencephalic neurons (MesV neurons), control the strength of occlusion and the position of the mandible. The mechanisms underlying cortical processing of proprioceptive information are critical to understanding how sensory information from the masticatory muscles regulates orofacial motor function. However, these mechanisms are mostly unknown. The present study aimed to identify the regions that process proprioception of the jaw-closing muscles using *in vivo* optical imaging with a voltage-sensitive dye in rats under urethane anesthesia. First, jaw opening that was produced by mechanically pulling down the mandible evoked an optical response, which reflects neural excitation, in two cortical regions: the most rostroventral part of the primary somatosensory cortex (S1) and the border between the ventral part of the secondary somatosensory cortex (S2) and the insular oral region (IOR). The kinetics of the optical signal, including the latency, amplitude, rise time, decay time and half duration, in the S1 region for the response with the largest amplitude were comparable to those in the region with the largest response in S2/IOR. Second, we visualized the regions responding to electrical stimulation of the masseter nerve, which activates both motor efferent fibers and somatosensory afferent fibers, including those that transmit nociceptive and proprioceptive information. Masseter nerve stimulation initially excited the rostral part of the S2/IOR region, and an adjacent region responded to jaw opening. The caudal part of the region showing the maximum response overlapped with the region responding to jaw opening, whereas the rostral part overlapped with the region responding to electrical stimulation of the maxillary and mandibular molar pulps. These findings suggest that proprioception of the masseter is processed in S1 and S2/IOR. Other sensory information, such as nociception, is processed in a region that is adjacent to these pulpal regions and is located in the rostral part of S2/IOR, which receives nociceptive inputs from the molar pulps. The spatial proximity of these regions may be associated with the mechanisms by which masseter muscle pain is incorrectly perceived as dental pain.

## Introduction

Muscle spindles in the jaw-closing muscles detect the length and tension of these muscles. This sensory information may play a pivotal role in regulating the strength of occlusion and the position of the mandible, which enables us to perform mastication and produce accurate speech. The neurons in the trigeminal mesencephalon (MesV) are the primary neurons that process sensory information from the muscle spindles in the jaw-closing muscles, which include the masseter and temporalis muscles (Lennartsson, 1980). The MesV neurons encode the distance between the mandible and maxilla (Yamamoto et al., 1989; Masri et al., 2005) and the velocity of jaw movement (Masri et al., 2005). MesV neurons project to the trigeminal motor nucleus (TMN) and process information involving the motor neurons that innervate jaw-opening and jaw-closing muscles (Dessem and Taylor, 1989; Luo et al., 1991; Luo and Dessem, 1995). The jaw stretch reflex is mediated by a monosynaptic pathway from muscle spindles to motor neurons (Luo and Li, 1991; Dessem et al., 1997; Luo et al., 2001).

In addition to the TMN, MesV neurons in the rat project to the trigeminal nuclei, which include the supratrigeminal region (Vsup), the dorsomedial part of the trigeminal principal sensory nucleus (Vpdm), the dorsomedial part of the trigeminal spinal nucleus oralis (Vodm), the dorsomedial part of the spinal trigeminal nucleus interpolaris (Vidm), nucleus caudalis of the spinal trigeminal nucleus (Vc) and the parvicellular reticular formation (PCRt; Luo et al., 1991; Luo and Dessem, 1995). Among these nuclei, closely apposed contacts from MesV neurons that target trigeminothalamic neurons are principally observed in the caudolateral part of Vsup (12%–20%); some contacts are present in the Vpdm, Vidm and PCRt (<3%), but no direct contacts are present in the Vc (Luo and Dessem, 1995). Thus, it is likely that in rats, the sensory information from jaw-closing muscles is conveyed to the cerebral cortex via these thalamic nuclei.

Although proprioceptive information originating in the muscle spindles is considered to be processed in the cerebral cortex, the locations and temporal properties of the cortical neurons responding to stimulation of these muscle spindles in the rat are relatively unknown. Electrical stimulation of the intercostal muscles elicits neural activity in the somatosensory areas of the human brain (Gandevia and Macefield, 1989). In the cat, selective activation of respiratory muscle mechanoreceptors elicits neural excitation in area 3a of the sensorimotor cortex (Davenport et al., 1993). Iwata et al. (1985) also demonstrated neuronal activity elicited by stimulation of the masseter nerve in areas 3a, 3b and 6aβ of cats. The orofacial somatosensory regions of rats are distributed in the primary (S1) and in secondary (S2) somatosensory cortex and dorsal insular cortex which is called the insular oral region (IOR; Remple et al., 2003; Nakamura et al., 2015). Considering the functional differences among these areas, understanding the spatiotemporal profiles of cortical excitation elicited by stimulating muscle spindles is critical to elucidating the mechanisms of cortical processing of proprioceptive information.

The somatotopic organization of the orofacial structures in rat cerebral cortex was previously demonstrated using field potential recordings (Remple et al., 2003) and optical imaging (Horinuki et al., 2015, 2016; Nakamura et al., 2015, 2016). These studies demonstrated that electrical stimulation of the whisker pad, mentum, tongue, dental pulps and periodontal ligaments induced cortical excitation in the ventral part of S1 and S2 and in the IOR. Therefore, muscle spindles in the jaw-closing muscles, which are the orofacial components, may induce excitation in similar regions of S1 and S2/IOR. However, it is unclear whether the sensory information from jaw-closing muscles is represented in the orofacial somatosensory regions.

Optical imaging using a voltage-sensitive dye enables us to visualize the spatial pattern of cortical excitation with a higher resolution than possible from recording field potentials. In the present study, we performed *in vivo* optical imaging with a voltage-sensitive dye in rats under urethane anesthesia to explore the specific cortical regions that respond to muscle spindle stimulation with temporal information.

## Materials and Methods

The experiments were approved by the Animal Experimentation Committee of Nihon University and were performed in accordance with the institutional guidelines for the care and use of experimental animals described in the National Institute of Health *Guide for the Care and Use of Laboratory Animals*. All efforts were made to minimize animal suffering and to reduce the number of animals used. We bought and used Sprague-Dawley rats which were breeding in Sankyo Labo. In our study, the vulnerable populations were not involved.

### *In Vivo* Optical Imaging

We performed optical imaging using a voltage-sensitive dye (RH1691, Optical Imaging, New York, NY, USA) as previously described (Kobayashi et al., 2010; Fujita et al., 2011, 2012, 2016; Horinuki et al., 2015, 2016; Nakamura et al., 2015, 2016). It is well established that the optical signal intensity correlates to the membrane potential of neurons and that changes in the membrane potential, including excitatory and inhibitory postsynaptic potentials, can be estimated by measuring the intensity of optical signals in real time (Berger et al., 2007; Chemla and Chavane, 2010; Fujita et al., 2010, 2011). Therefore, an increase in the optical signal using RH1691 is regarded as excitation of neurons (Petersen, 2006). Six- to eight-week-old male Sprague-Dawley rats (Sankyo Labo, Tokyo, Japan) weighing 205.3 ± 9.1 g (*n* = 18) received an injection of atropine methyl bromide (5 mg/kg, i.p.) and were anesthetized with urethane (1.5 g/kg, i.p.). The efficacy of anesthesia was gauged by the toe pinch reflex, and additional urethane was administered as needed. Body temperature was monitored using a rectal probe and was maintained at approximately 37°C using a heating pad (BWT-100, Bio Research Center, Osaka, Japan). A tracheotomy and intubation were performed. Lidocaine (2% gel, AstraZeneca, Tokyo, Japan) was administered to the incisions to ensure complete analgesia. The animal was fixed to a custom-made stereotaxic snout frame, which was tilted 60° laterally for imaging the surface of the left insular cortex using a CCD camera (MiCAM02, Brainvision, Tokyo, Japan). The left temporal muscle and zygomatic arch were carefully removed, and a craniotomy was performed to expose the insular and surrounding cortices.

RH1691 (1 mg/ml) was dissolved in 0.9% saline and applied to the cortical surface for 1 h. Changes in RH1691 fluorescence were measured using the CCD camera system described above, which was mounted on a stereomicroscope (Leica Microsystems, Wetzlar, Germany). The cortical surface was illuminated through a 632-nm excitation filter and a dichroic mirror using a tungsten-halogen lamp (CLS150XD, Leica Microsystems). The fluorescent emission was captured through an absorption filter (*λ* > 650-nm longpass, Andover, Salem, MA, USA). The CCD camera had a 6.4 mm^2^ × 4.8 mm^2^ imaging area (184 × 124 pixels).

To remove signals due to acute bleaching of the dye, values in the absence of any stimuli were subtracted from each recording: each image was constructed from paired recordings with and without stimulation. The sampling rate was set at 250 Hz, and the acquisition time was 500 ms. Forty consecutive images in response to the stimuli were averaged to reduce the noise described above.

### Stimulation of Muscle Spindles and Dental Pulps

To stretch the muscle spindles in jaw-closing muscles, the mandible was tied with a wire at the frontal part of the masseter muscle. The jaw was opened by pulling the wire downward using a motor unit (Solar Motor 03, Tamiya, Shizuoka, Japan) for 10 ms. This stimulation protocol induced only a slight jaw opening, which induced less mechanical noise than more pronounced jaw opening but was sufficient to induce cortical responses. A small rubber band was used to return the jaw to the original position.

**Figure 1:**
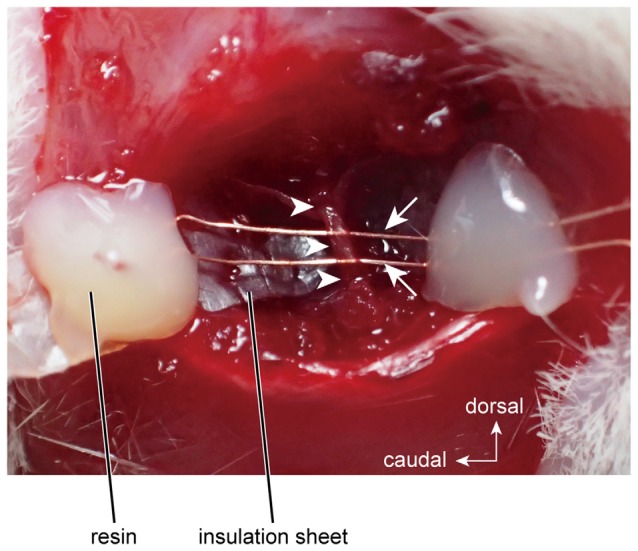
**Electrical stimulation of the masseter nerve using bipolar electrodes.** Arrows and arrowheads indicate the stimulation electrodes and the masseter nerve, respectively.

The right masseter nerve was mounted on bipolar electrodes, which were made from enamel-coated copper wire (diameter = 80 μm; Tamagawa-densen, Tokyo, Japan; Figure 1), and was electrically stimulated with a rectangular pulse (3–7 V, 100-μs duration) by a stimulator (STG2008, Multi-Channel Systems, Reutlingen, Germany).

The maxillary and mandibular 1st molar pulps were electrically stimulated with a bipolar electrode as previously described (Nakamura et al., 2015, 2016). Five voltage pulses (3 V, 100-μs duration) were applied at 50 Hz for dental pulp stimulation. The stimulation intervals were set at 20 s to obtain stable optical responses. To ensure that results in this study were comparable to our previous results (Nakamura et al., 2015, 2016), we applied 5 voltage pulses in molar pulp stimulation.

### Anatomy

After optical imaging, the regions within S1 and S2/IOR that exhibited responses were marked by penetration with a heated needle. The rats were deeply anesthetized with 5.0% isoflurane and perfused through the ascending aorta with saline and then by 300 ml of a fixative containing 4% paraformaldehyde in 0.1 M phosphate buffer (PB, pH = 7.4). The brains were removed, post-fixed overnight and cryoprotected in 30% sucrose in 0.1 M PB. The brains were frozen, coronally sectioned at 50 μm, and stained with 0.25% thionine for histological examination of the responding regions. S1 and S2 were defined according to a rat brain atlas (Paxinos and Watson, 2007). Nissl-stained sections also show that the claustrum is present between the white matter and the insular cortex but not S1 and S2 (Kobayashi, 2011). In addition, cytochrome oxidase-stained flat-mount sections helped us distinguish S2 from S1 and insular cortex (Remple et al., 2003; Nakamura et al., 2015).

### Data Analysis

**Figure 2:**
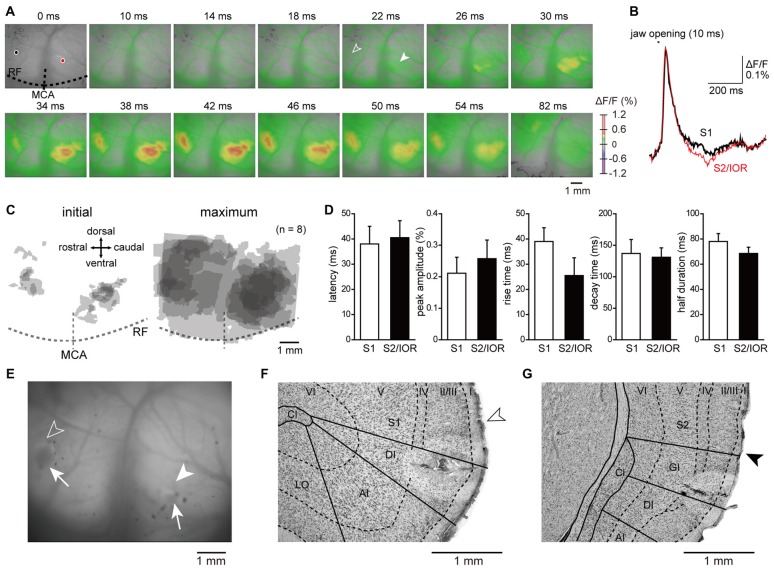
**Jaw opening elicited excitatory propagation in the primary somatosensory cortex (S1) and secondary somatosensory cortex (S2)/insular oral region (IOR) that was revealed by optical imaging. (A)** An example of excitatory propagation elicited by jaw opening (10 ms). The amplitude of ΔF/F was color coded, and the time from the onset of jaw opening is shown at the top of each panel. Outlined and solid arrowheads indicate the initial responses in S1 and S2/IOR, respectively. Note that the excitation was found not only in S1 but also in S2/IOR, which is around the insular cortex and S2 and caudal to the middle cerebral artery (MCA). **(B)** The temporal profiles of optical signals in the regions of interest are indicated by black circles (S1) and red circles (S2/IOR) in **(A)**. **(C)** The superimposed images of the initial and maximum responses in reference to the MCA and rhinal fissure (RF). The number of overlapping responses is represented by the gradation of colors. **(D)** The latency, peak amplitude, rise time, decay time and half duration of excitation in S1 and S2/IOR (*n* = 8). **(E)** The coagulated points (arrows) and the initial responses to jaw opening in S1 (outlined arrowhead) and S2/IOR (solid arrowhead). The coagulated points were made by penetration with a heated needle after experiments as landmarks to confirm the responding regions. **(F,G)** Coronal Nissl sections showing the coagulated points and the initial responses in S1 (open arrowhead, **F**) and S2/IOR (closed arrowhead, **G**). Note that the data shown in **(A,B**,**E–G)** were obtained from the same animal. AI, agranular part of insular cortex; Cl, claustrum; DI, dysgranular part of insular cortex; GI, granular part of insular cortex; LO, lateral orbital cortex.

Changes in the intensity of fluorescence (ΔF) of each pixel relative to the initial intensity of fluorescence (F) were calculated (ΔF/F), and the ratio was processed with a spatial filter (9 × 9 pixels). A significant response was defined as a signal exceeding seven times the SD of the baseline noise, as previously described (Nakamura et al., 2015, 2016). The optical imaging data were processed and analyzed using Brain Vision Analyzer software (Brainvision, Tokyo, Japan). Images were aligned across multiple rats using the rhinal fissure (RF) and middle cerebral artery (MCA) as markers. In 4% of the rats, the MCA exhibited angioplany, e.g., it was bifurcated at the RF. In these animals, the RF and the MCA could not be aligned with the other animals; therefore, we excluded the results obtained from these animals. We estimated the spatial profiles of excitation using the initial and maximum responses (Figure 2). The initial response was obtained by outlining the excitation evoked in the first frame that exhibited a significant increase in the optical signal. The maximum response was defined as the outline of the excitatory response in the frame with the maximum amplitude of the optical signal in the center of the initial response. In our observations, jaw opening achieved by pulling the wire tied to the jaw and jaw closing achieved by masseter nerve stimulation elicited positive and negative changes, respectively, in the optical intensity in the rostral part of the RF, which was located in the lower-left region of the observed area. We considered these responses as artificial signals due to jaw movements and excluded these changes in optical intensity from the present analyses.

In this study, we defined the latency as the time elapsed between the onset of stimulation and the time at which a significant optical response was first detected. The peak amplitude was the maximum amplitude of an optical response at the point of the initial response. The rise time was the duration from the onset of the optical response to the maximum response, whereas the decay time was the duration from the maximum response to the return to baseline. The half duration was the duration at half maximum. In some cases, there was no significant response observed in S1. We excluded these cases from comparisons of latency, rise time, decay time and half duration.

### Statistics

The data are expressed as the mean ± SEM. Paired *t* tests were used in the analyses. Values of *P* < 0.05 were considered significant. In multiple comparisons, we applied the Bonferroni correction with a Bonferroni-corrected probability value of *P* < 0.025 considered statistically significant.

## Results

In rats, there are a number of muscle spindles in jaw-closing muscles including the masseter, temporalis and medial pterygoid muscles, whereas few muscle spindles exist in jaw-opening muscles (Lennartsson, 1980). The optimal mechanical stimulation of muscle spindles is stretching the muscle that involves them (Davenport et al., 1993), and therefore, in the first series of experiments, we applied jaw opening to activate the muscle spindles in jaw-closing muscles.

In the second series of experiments, we used electrical stimulation of the masseter nerve, because jaw opening activates not only muscle spindles in jaw-closing muscles but also mechanoreceptors in the temporal mandibular joint (Kawamura and Abe, 1974). Furthermore, in comparison to mechanical stimulation, electrical stimulation provides precise temporal activation of the spindle afferents. The masseter includes the greatest number of muscle spindles among jaw closing muscles (Lennartsson, 1980) and proprioceptive information from muscle spindles in the masseter is conveyed via the masseter nerve. Although the masseter nerve contains nociceptive fibers (Nishimori et al., 1986; Shigenaga et al., 1988; Ro et al., 2003), electrical stimulation of the masseter nerve effectively activates muscle spindle afferents.

### Cortical Regions Responding to Jaw Opening

To identify the cortical region that mediates proprioception of the jaw-closing muscles, optical signals in the insular and the surrounding cortices, including S1 and S2, were imaged using *in vivo* preparations. Jaw opening elicited an early neural excitation in the rostroventral part of S1 and the border between the ventral part of S2 and IOR (Figure 2). Then, the neural excitation spread to the surrounding regions. The kinetics of the optical response, including the latency, peak amplitude, rise time, decay time and half duration, were examined in the center of the initial response observed in S1 and S2/IOR (see “Materials and Methods” Section). These parameters of kinetics were comparable between S1 and S2/IOR. These results suggested parallel processing of sensory information associated with jaw opening in S1 and S2/IOR.

### Cortical Responses Evoked with Electrical Stimulation of the Masseter Nerve

**Figure 3:**
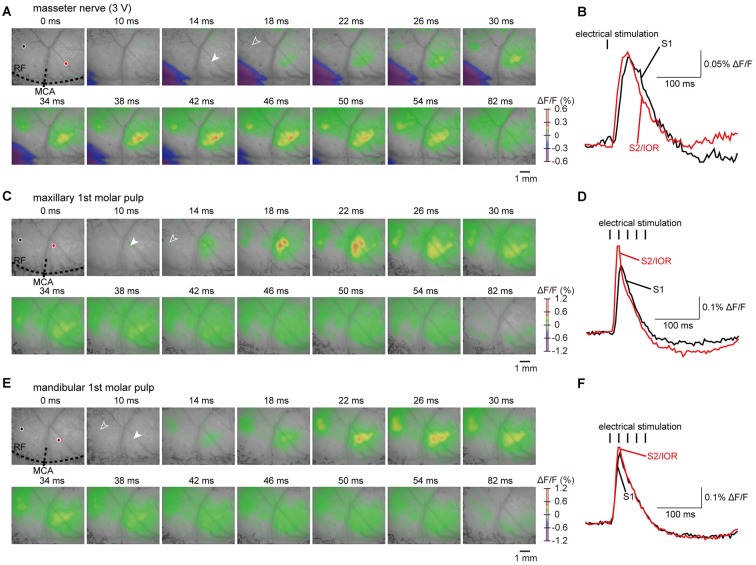
**Examples of cortical responses evoked by electrical stimulation of the masseter nerve and molar pulps. (A,C,E)** The S1 and S2/IOR responses evoked by electrical stimulation of the masseter nerve **(A)** and maxillary **(C)** and mandibular 1st molar pulps **(E)**. The outlined and solid arrowheads indicate the initial responses in S1 and S2/IOR, respectively. The time from the onset of electrical stimulation is shown at the top of each panel. **(B,D,F)** The temporal profiles of optical signals in S1 (black) and S2/IOR (red) are indicated by black and red circles in **(A,C,E)**, respectively.

**Figure 4:**
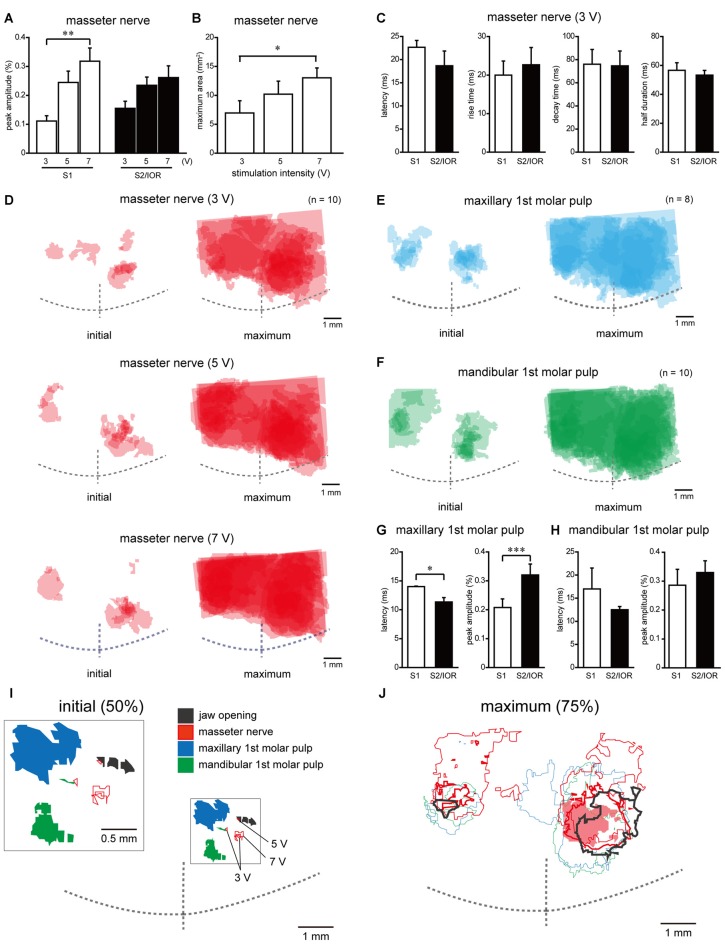
**Quantitative analyses of spatiotemporal profiles of cortical responses evoked by electrical stimulation of the masseter nerve and molar pulps. (A)** The stimulation intensity-dependent peak amplitude in S1 and S2/IOR evoked by masseter nerve stimulation (*n* = 10; **P < 0.005, paired *t* test with Bonferroni correction). **(B)** The stimulation intensity-dependent maximum area evoked by masseter nerve stimulation (*n* = 10; *P < 0.025, paired *t* test with Bonferroni correction). **(C)** Comparison of the latency, rise time, decay time and half duration of excitation between the S1 and S2/IOR regions responding to electrical stimulation of the masseter nerve at 3 V (*n* = 6). **(D–F)** The superimposed images of the initial and maximum responses evoked with masseter nerve **(D)** and maxillary **(E)** and mandibular 1st molar pulp **(F)** stimulation. The number of overlapping responses is represented by the gradation of colors. ** (G,H)** Comparison of the latency and peak amplitude of excitation response evoked with maxillary **(G)** and mandibular 1st molar pulp stimulation **(H)** between S1 and S2/IOR (*n* = 8–10; *P < 0.05, ***P < 0.001, paired *t* test).** (I)** The spatial distribution of the initial response that overlapped in 50% of the rats. Note that areas that responded to jaw opening and masseter nerve stimulation are located caudal to the area that showed responses to maxillary and mandibular 1st molar pulp stimulation. An inset shows magnified scheme in the box. **(J)** The spatial distribution of the maximum responses, which overlapped in 75% of the rats. The red shaded area, thick and thin lines indicate outlines of responses to electrical stimulation of masseter nerve at 3 V, 5 V and 7 V, respectively. Note that the area responding to masseter nerve stimulation expanded rostrally compared to the area responding to jaw opening.

It is reasonable to postulate that the cortical responses observed with jaw opening were elicited by the excitation of muscle spindles in only the jaw-closing muscles because few muscle spindles are present in the jaw-opening muscles, such as the digastric muscles, in rats (Lennartsson, 1980). To examine this hypothesis, we then imaged the responses to electrical stimulation of the masseter nerve, which innervates the masseter muscle. Stimulation of the masseter nerve evoked neural excitation that was similar to the response observed with jaw opening (Figure 3A). We applied the electrical stimulation at an intensity that ranged from 3 V to 7 V (Figure 4; *n* = 10).

The initial responses were observed in the rostroventral part of S1 and the ventral part of S2 (Figures 3A,B). The neural excitation then spread concentrically to the surrounding regions. S2/IOR was consistently excited by masseter nerve stimulation applied at 3–7 V. On the other hand, S1 showed faint excitation to stimulation applied at 7 V (9/10 animals), 5 V (7/10 animals) and 3 V (6/10 animals). In the present study, we quantitatively analyzed the excitatory propagation evoked with stimulation at 3 V and then excluded cases in which the S1 responses were faint in comparison to the temporal kinetics of excitation observed between S1 and S2/IOR.

The peak amplitude showed a stimulation intensity-dependent increase in S1 (*P* < 0.005, paired *t* test with Bonferroni correction; Figure 4A). S2/IOR also showed a similar tendency, although this effect was not significant. The latency, rise time, decay time and half duration were not different between S1 and S2/IOR (Figure 4C).

In addition to the ROI analyses, the maximum area (see “Materials and Methods” Section) was analyzed. The maximum area expanded in a stimulation intensity-dependent manner (Figures 4B,D; *P* < 0.025, paired *t* test with Bonferroni correction).

In a subset of the experiments, the recording was performed before and after cutting the masseter nerve at the peripheral side of the stimulation electrode to examine the possibility that the evoked cortical excitation may respond to the masseter muscle movement. Although the cortical responses to the transected nerve stimulation were smaller in amplitude than the responses to the intact nerve stimulation, their spatiotemporal profiles were comparable (data not shown). This finding suggests that the major part of the cortical responses to the masseter nerve stimulation is responses via electrically stimulated afferents but not responses to jaw movements mediated by efferents.

### Regions Responding to Masseter Nerve Stimulation in Reference to Regions Responding to Dental Pulp Stimulation

To identify the anatomical landmarks for regions responding to masseter nerve stimulation, we imaged the regions responding to electrical stimulation of the maxillary and mandibular 1st molar pulps. Representative examples obtained from the same animal represented in Figures 3A,B are shown in Figures 3C–F. In agreement with our previous observations (Nakamura et al., 2015, 2016), the initial response to stimulation of the maxillary 1st molar pulp was observed in S2/IOR (Figures 3C,D). The anatomical location was immediately caudal to the MCA (Figures 3C, 4E). The initial response to stimulation of the mandibular 1st molar pulp was observed in the ventral part of the region that showed the initial response to stimulation of the maxillary 1st molar pulp (Figures 3E, 4F). The latency and peak amplitude of the excitation evoked in S2/IOR with stimulation of the maxillary 1st molar pulp was shorter and higher than those observed in S1 (*P* < 0.05, paired *t* test), whereas the kinetics of the excitation evoked with stimulation of the mandibular 1st molar pulp in S1 were comparable to those in S2/IOR (Figures 4G,H).

To elucidate the spatial distribution patterns of the regions responding to jaw opening, masseter nerve stimulation and dental pulp stimulation, the outlines of their initial and maximum responding regions in 50% and 75% of animals were merged in reference to the crosspoint of the RF and the MCA (Figures 4I,J). The initial response to jaw opening was caudal to the region that responded to stimulation of the maxillary 1st molar pulp. Electrical stimulation of the masseter nerve at 3–7 V evoked an initial response between the region responding to jaw opening and the region responding to stimulation of the mandibular 1st molar pulp.

On the other hand, some of the maximum responses evoked with jaw opening or stimulation of the maxillary or mandibular 1st molar pulp or the masseter nerve overlapped in S1 and S2/IOR (Figure 4J). The excitation area in maximum response to masseter nerve stimulation showed an intensity-dependent increase, and the area responding to masseter nerve stimulation at 7 V expanded dorsally. However, the outline of the region responding to masseter nerve stimulation was biased toward the region responding to molar pulp stimulation compared to that for jaw opening.

### Short-Term Plasticity of the S2/IOR Response Evoked with Masseter Nerve Stimulation

**Figure 5:**
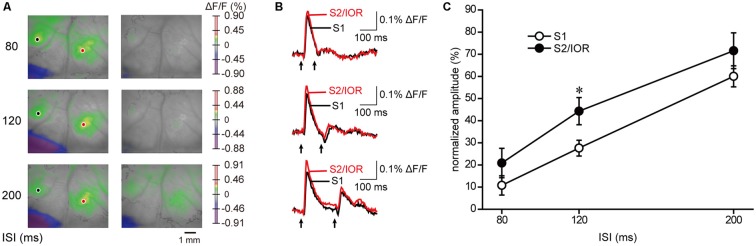
**Short-term plasticity in response to masseter nerve stimulation. (A)** Examples of the maximum response to the 1st and 2nd stimuli. The interstimulus interval (ISI) was set at 80 ms, 120 ms or 200 ms. Paired-pulse depression was observed.** (B)** The temporal profiles of optical signal amplitudes in the region of interest in S1 (black) and S2/IOR (red) in **(A)**. Paired-pulse stimulation is indicated by arrows. **(C)** A faster recovery of the second response in S2/IOR than in S1 at 120 ms ISI (*n* = 6; *P < 0.05, paired *t* test).

Jaw opening and electrical stimulation of the masseter nerve evoked comparative kinetics of excitation, including the latency, amplitude, rise time and decay time and half duration, in S1 and S2/IOR (Figures 2, 4). However, whether S1 and S2/IOR process the information in a completely similar manner remained unclear. To further explore the characteristics of the processing systems in S1 and S2/IOR, we examined short-term plasticity by applying paired-pulse stimulation (Figure 5). The interstimulus interval (ISI) was set at 80 ms, 120 ms or 200 ms. Paired-pulse depression was observed at each of these ISIs, and gradual recovery was observed when the ISI was longer. With a 200-ms ISI, the ratios of recovery in S1 and S2/IOR were 60% and 72%, respectively. The ratio in S2 was higher than that in S1 at 120 ms (*P* < 0.05, paired *t* test). This result indicates that S1 and S2/IOR have different neural systems.

## Discussion

In the present study, we show the spatial distribution patterns of excitation in S1 and S2/IOR elicited by jaw opening, masseter nerve stimulation and maxillary or mandibular molar pulp stimulation. In S2/IOR, the initial response to jaw opening was observed in the region caudal to the region that showed excitation in response to molar pulp stimulation. Compared to the area responding to jaw opening, the region responding with excitation to masseter nerve stimulation in a maximum response expanded to a region that was rostral to the region excited by dental pulp stimulation. These findings suggest that proprioceptive information from the masseter is processed in S1 and S2/IOR, in regions adjacent to those processing information from dental pulp. Overlapping S2/IOR regions responding to masseter nerve and molar pulp stimuli may contribute to the mechanisms through which muscle pain in the masseter is incorrectly perceived as molar pain (Bender, 2000; Wright, 2000).

### Jaw Opening vs. Masseter Nerve Stimulation

In the present study, we observed responses to jaw opening and masseter nerve stimulation in the most rostroventral parts of S1 and S2/IOR. In the cat, masseter nerve stimulation elicits neural excitation in 3a, 3b and 6aβ (Iwata et al., 1985), suggesting that proprioceptive information from muscle spindles is processed in the area between the somatosensory and motor cortices. Considering the patterns observed in the Nissl stained-sections, the region around S1 that showed excitation likely corresponds to areas 3a, 3b and 6aβ. The present study focused on the spatiotemporal responses in S2/IOR because S1 often shows inconsistent responses.

Jaw-closing muscles involve a number of muscle spindles which are activated by stretching, e.g., jaw opening (Lennartsson, 1980; Davenport et al., 1993). The masseter nerve conveys proprioceptive information from muscle spindles to central nervous system. In this study, we applied jaw opening and electrical stimulation of the masseter nerve, which commonly stimulates muscle spindle afferents in the masseter, a primary jaw-closing muscle. As a result, the overlapped region in S2/IOR that was activated by jaw opening and also by masseter nerve stimulation (Figures 2, 3) is likely to mediate proprioception of the masseter muscle. Electrical stimulation of the masseter nerve induced less activation in the caudal part of S2/IOR compared to the activated region responding to jaw opening (Figure 4J). This discrepancy of the activated regions between electrical and mechanical stimulation may be due to the differences in the activated sensory fibers as follows. Jaw opening activates the muscle spindles not only in the masseter but also those in the temporalis and medial pterygoid muscles. According to our previous study that transcallosal fibers are symmetrically connected in the somatosensory and insular cortices (Fujita et al., 2012), jaw opening is likely to activate more cortical neurons by stretching the muscle spindles in both sides of the closing muscles.

In contrast to the decreased activation in the caudal part of S2/IOR, electrical stimulation of the masseter nerve additively activated the rostral part (Figure 4J). The region responding only to electrical stimulation might be activated by nociceptive inputs. In addition to proprioceptive afferents, the masseter nerve also contains nociceptive afferents, which terminate in the trigeminal sensory complex in cats (Nishimori et al., 1986; Shigenaga et al., 1988), and masseter muscle inflammation produces *c-fos* expression in the trigeminal sensory nuclei of rats (Ro et al., 2003). This nociceptive information might elicit patterns of neural excitation that differ between jaw opening and masseter nerve stimulation. First, the initial response to the masseter nerve was observed between the region responding to jaw opening and the region responding to mandibular 1st molar pulp stimulation (Figure 4I). Second, the area responding to masseter nerve stimulation expanded rostrally (Figure 4J). This expansion might not have been due to the difference in the stimulation intensity required to drive neural excitation. The peak amplitude of the masseter nerve stimulation at 3 V was smaller in S1 and S2/IOR than that observed with one instance of jaw opening, and the area of the maximum response in S1 was smaller for masseter nerve stimulation than for one instance of jaw opening. Although these results indicate that masseter nerve stimulation at 3 V induced a small amount of neural excitation compared to jaw opening, the maximum response to masseter nerve stimulation at 3 V showed an area that expanded into the rostral region of the area that initially responded to dental pulp stimulation (Figure 4J). Thus, nociceptive information from the masseter might contribute to an expanded area of neural activity evoked with masseter nerve stimulation. The threshold to elicit excitation of neural fibers is dependent on their diameter: stimulation at low and high intensities causes the activation of A and C fibers, respectively (Takemura et al., 2000; Fukui et al., 2007; Fujisawa et al., 2012). This finding suggests the possibility that electrical stimulation at low intensity might function to isolate fibers of the masseter nerve transmitting nociceptive and proprioceptive information. However, only faint responses were obtained by stimulation of the masseter nerve at <3 V, and those responses could not satisfy the criteria for reliable analysis of the optical signals. In addition to an involvement of nociceptive inputs, electrical stimulation may activate secondary muscle spindle afferent fibers (II) in addition to the primary afferent fibers (Ia). Although rapid muscle stretch activates both types of the spindle afferents (Botterman and Eldred, 1982; Fitz-Ritson, 1982), Ia and II afferents are sensitive to the rate of change of stretch and to the middle to maximum muscle lengths, respectively. This study applied small jaw opening with a short duration, and therefore, jaw opening is likely to activate principally Ia but not II afferents. If it is the case, the rostral region activated by electrical stimulation of the masseter muscle may receive inputs from II afferents.

In alert mice, excitation in the primary somatosensory (barrel) cortex elicited by whisker deflection is followed by activation of the whisker motor cortex via cortico-cortical connections (Ferezou et al., 2007; Aronoff et al., 2010; Feldmeyer et al., 2013). This subsequent excitation in the motor cortex could be applicable to the proprioceptive information processing. Indeed, the optical responses were initially elicited in the rostroventral part of S1 and the border between the ventral part of S2 and IOR, and spread to the dorsal area involving a part of the motor cortex. The excitation in the dorsal area continued even after elimination of the S1 response (see at 82 ms after the onset of stimulation in Figures 2A, 3A). Similarly, the responses to electrical stimulation of the masseter nerve at 7 V expanded the maximum response to the dorsal area (Figure 4J). These findings imply the possibility that the somatosensory cortical excitation induces excitation in the motor-related area (Adachi et al., 2008).

### Functional Implications

We can easily detect a very small fraction of a distance, such as the width of a hair (*φ* = 50–150 μm), between the maxillary and mandibular teeth. This suggests that the oral mechanosensory organs in the periodontal ligament and muscle spindles in the jaw-closing muscles have a greater sensitivity than those in the skin (Lund and Kolta, 2006). Indeed, it is known that muscle spindles are highly sensitive and respond to low-amplitude stretching. The spindles in the intercostal muscles of cats are activated with an approximate 15-μm stretch, and 94% of the muscle spindles are stimulated with a 300-μm stretch (Bolser et al., 1987). The amplitude of cortical evoked potentials in area 3a of the sensorimotor cortex in cats can reach 20% of the maximum response with a 50-μm stretch of intercostal space (Davenport et al., 1993). Thus, small mechanical stimulation is sufficient to stimulate muscle spindles.

In contrast, we often cannot identify the precise region of nociception in the oral cavity, e.g., misunderstanding of a diseased tooth and referred pain (nociception in the orofacial region frequently causes the perception of pain in other regions; Bender, 2000; Wright, 2000). Because most of the nerve fibers in the dental pulp consist of Aδ and C fibers, it is considered that the dental pulp alone transmits nociceptive information (Shigenaga et al., 1974; Belforte and Pazo, 2005). Our previous studies have demonstrated that early responses (10–18 ms after stimulation) to stimulation of the maxillary or mandibular molar pulp or of the periodontal ligaments are found at separate locations in S2/IOR, but the maximum responses, which occur approximately 30 ms after stimulation, occur in regions that almost overlap (Horinuki et al., 2015, 2016; Nakamura et al., 2015, 2016). These findings imply a poor capacity for spatial identification of nociception in the oral region.

The proximity of the cortical regions that responded to masseter nerve stimulation and molar pulp stimulation in S2/IOR suggests the possibility that nociception of the masseter muscle may be incorrectly perceived as pulpal nociception. Indeed, clinically, it is well known that the masseter muscle is one of the most common sources of referred pain in the craniofacial region, including pain originating in the maxillary and mandibular molars (Wright, 2000). The present findings may be one of the underlying mechanisms for the mislocalization of orofacial pain. The new hypotheses regarding the area processing nociceptive information from the masseter, which might contribute to referred pain, and regarding the proprioception-related excitation in the motor cortex should be addressed in future research.

## Author Contributions

MKo designed the research; MKa and HN performed the research; SF and HN analyzed the data; SF and MKo wrote the article.

## Funding

This work was supported by the Japan Society for the Promotion of Science KAKENHI (25293379 to MKo); The Ministry of Education, Culture, Sports, Science and Technology-Supported Program for the Strategic Research Foundation at Private Universities, 2013–2017, to MKo; The Science Research Promotion Fund from The Promotion and Mutual Aid Corporation for Private Schools of Japan to SF; and the Sato Fund and Dental Research Center at Nihon University School of Dentistry.

## Conflict of Interest Statement

The authors declare that the research was conducted in the absence of any commercial or financial relationships that could be construed as a potential conflict of interest.
